# The Understanding of Visual Metaphors by the Congenitally Blind

**DOI:** 10.3389/fpsyg.2018.01242

**Published:** 2018-07-23

**Authors:** Ricardo A. Minervino, Alejandra Martín, L. Micaela Tavernini, Máximo Trench

**Affiliations:** ^1^Department of Psychology, Instituto Patagónico de Estudios de Humanidades y de Ciencias Sociales–Consejo Nacional de Investigaciones Científicas y Técnicas, University of Comahue, Neuquén, Argentina; ^2^Institute of Economic and Social Research of the South, Universidad Nacional del Sur–Consejo Nacional de Investigaciones Científicas y Técnicas, Bahía Blanca, Argentina; ^3^Department of Psychology, Instituto Patagónico de Estudios de Humanidades y de Ciencias Sociales–Consejo Nacional de Investigaciones Científicas y Técnicas, University of Comahue, Bariloche, Argentina

**Keywords:** figurative language, metaphor, conceptual metaphor, embodiment, blind

## Abstract

Results from a narrow set of empirical studies suggest that blind individuals’ comprehension of metaphorical expressions does not differ from that of sighted participants. However, prominent accounts of metaphor comprehension yield different predictions about the blind’s ability to comprehend visual metaphors. While conceptual metaphor theory leads to predicting that blind individuals should lag behind their sighted peers in making sense of this particular kind of utterances, from traditional accounts of analogical reasoning it follows that blind individuals’ ability to comprehend the literal meaning of visual concepts might be sufficient to support their metaphorical application. In Experiment 1, 20 sighted and 20 congenitally blind participants were asked to select the most appropriate meaning for visual, grasping and filler metaphorical expressions. Results failed to reveal group differences for any type of metaphorical expressions. In order to implement a more stringent test of blind individuals’ ability to understand visual metaphors, in Experiment 2 blind and sighted participants were presented with very novel figurative expressions, as indicated by low or no occurrence in the “Google” corpus. In line with the results of Experiment 1, blind participants’ comprehension of visual metaphors was both high in absolute terms and comparable to that of sighted participants. We advance some speculations about the mechanisms by which blind individuals comprehend visual metaphors and we discuss the implications of these results for current theories of metaphor.

## Introduction

Initial studies looking at differences between blind and sighted populations in their comprehension of figurative language were primarily concerned with carefully assessing anecdotal reports of educators, according to which sensory deprivation might be associated with a delay in the acquisition of pragmatic abilities and of a theory of mind. In one study, Pring et al. (1998) had blind and sighted children aged 9–11 read 24 “strange stories" originally used by Happe (1994) as a more advanced and naturalistic test of theory of mind than the standard theory-of-mind tasks. As an example, the “sarcasm" situation tells that Tom had convinced Sarah to go on a picnic based on the prediction that it would be a lovely sunny day, but that counter to Tom’s prediction, it starts raining heavily right after beginning to unpack the food, to what Sarah exclaims: “Oh yes, a lovely day for a picnic alright!" When queried about the reasons for specific actions of the characters (e.g., Sarah’s remark), blind participants provided just as many mental justifications as the sighted participants. However, their justifications were significantly less accurate. In a more recent study, Pijnacker et al. (2012) exposed blind and sighted children aged 5–13 to a subset of Happe’s “strange situations" involving non-literal language, and which consisted of lies, white lies, jokes, figure of speech, and irony or sarcasm. In contrast to the previous study, no group differences were obtained. In the particular case of metaphor comprehension, authors like Pérez-Pereira and Conti-Ramsden (1999) suggest that eventual differences between populations tend to be overcome in late adolescence (see Sak-Wernicka, 2017, for a review). Even though the available studies failed to obtain a general advantage of sighted participants for making sense of non-literal language, from conceptual metaphor theory (Lakoff and Johnson, 1980, Lakoff and Johnson, 1999; Lakoff, 2008, 2014) it follows that the congenitally blind might show some difficulties for comprehending a particular type of figurative expressions, namely, those in which abstract concepts are metaphorically understood in terms of more concrete concepts referred to the visual domain.

According to conceptual metaphor theory, linguistic expressions such as *We are approaching Friday, We’ll soon reach the end of the winter*, or *He left the old days behind* demonstrate the existence of a conceptual metaphor in English speakers’ minds, that is, an analogy between the base domain of space and the target domain of time. With regards to psychological mechanisms, conceptual metaphor theory posits that conceptual metaphors are instrumental in both producing and interpreting metaphorical expressions (Lakoff, 1993). For example, making sense of a sentence such as *We are approaching Friday* supposes the use of the “ego moving” version of the TIME IS SPACE conceptual metaphor, by which the passage of time is depicted in terms of one’s own displacement along a straight line in which successive points represent moments in the future. This way, the amount of time that will need to elapse for a future moment to occur is represented as the distance the person has to advance in order to reach its corresponding point in the line.

The typical experimental procedure for assessing the use of conceptual metaphors during the interpretation of metaphorical expressions consists in determining whether reading expressions derived from a given conceptual metaphor facilitates the comprehension of subsequent expressions pertaining to the same conceptual metaphor, as compared to a control condition in which those target expressions were preceded by expressions pertaining to a mismatching conceptual metaphor. A wealth of empirical studies employing this procedure (e.g., Gentner and Boronat, 1991; Allbritton et al., 1995; Gibbs et al., 1997; McGlone and Harding, 1998; Boroditsky, 2000; Gentner et al., 2002; Langston, 2002; Thibodeau and Durgin, 2008) demonstrate that people employ conceptual metaphors to understand metaphorical expressions.

Classic cognitive science has tended to consider concepts as amodal and arbitrary symbols, whereas embodied perspectives on cognition have proposed that concepts consist of modal analogical representations (e.g., Barsalou, 2008) and, ultimately, on neural patterns of sensorimotor activity (e.g., Gallese and Lakoff, 2005; Lakoff, 2008). A number of neuroimaging and behavioral studies showed the involvement of sensorimotor areas in conceptual processing. For example, the recognition of words highly associated with auditory features (e.g., “telephone”) elicits activation in areas of the auditory association cortex that are associated to sound perception (Kiefer et al., 2008). Reading action-related words and sentences increases activation in cortical regions responsible for performing the relevant movements (Hauk et al., 2004; Tettamanti et al., 2005). Behavioral studies have provided further evidence for the idea that concepts are grounded in perception and action. To give an example, Pecher et al. (2003) participants verified verbally expressed facts involving one modality (*Leaves rustle*) more rapidly after verifying a fact involving the same modality (*Blenders make noise*) than after verifying a fact involving a different modality (*Cranberries are tart*).

Research on conceptual metaphors has revealed that whereas the base domains refer to the application of perceptual and motor processes to physical entities (e.g., walking forward), target domains typically refer to abstract processes and entities (e.g., the passage of time; Lakoff and Johnson, 1980, Lakoff and Johnson, 1999). The embodied approach has considered conceptual metaphors as a particular medium by which the cognitive system can provide a perceptual basis to abstract concepts (Kövecses, 2015), which have posed a serious challenge to the embodied view (Dove, 2016). In line with this view, it has been postulated that our understanding of metaphorical expressions derived from conceptual metaphors is embodied, in the sense of involving the simulation of the sensory-motor experiences that lie at their foundation (Lakoff and Johnson, 1980, Lakoff and Johnson, 1999; Gallese and Lakoff, 2005; Gibbs, 2005; Feldman, 2006; Lakoff, 2008, 2014; Johnson, 2010; Kövecses, 2016). Authors like Gibbs (2005) or Johnson (2005) have emphasized that the projection of the sensory-motor content onto a target concept entails reproducing the qualitative subjective experience that characterizes the base domain, and not merely the transfer of an abstract and amodal schema devoid of phenomenological substrate. According to Gibbs (2006a), these sensory-motor simulations of the base domain are key to reaching an emphatic representation of the qualitative experience that the author of a metaphorical utterance has intended to communicate. Hence, interpreting a sentence like *He left the old days behind* requires simulating the experience of walking forward on a linear path while leaving physical objects behind.

Conceptual metaphor theory distinguishes between two kinds of conceptual metaphor: *primary* and *complex* (Grady et al., 1996; Lakoff and Johnson, 1999). Primary conceptual metaphors derive directly from our common bodily experience, whereas complex metaphors are combinations of primary metaphors and cultural beliefs. Primary conceptual metaphors like AFFECTION IS WARMTH—as conveyed in expressions such as *They gave me a warm welcome*—are believed to arise from correlations in everyday experiences where judgments about affective behavior are recurrently associated with sensory-motor experiences of physical warmth (e.g., as a consequence of being cuddled by a loving caregiver). From the neural perspective of conceptual metaphor theory (Lakoff, 2008), this co-activation causes the strengthening of connections between the neural circuits supporting different experiences, connections that become reactivated during the interpretation of metaphorical expressions.

In contrast to the wealth of evidence demonstrating the activation of conceptual metaphors during the interpretation of metaphorical expressions, only a handful of studies aimed at demonstrating the role of sensory-motor simulations in making sense of such expressions. For example, Wilson and Gibbs (2007) demonstrated that performing an action corresponding to a base concept (e.g., grasping) facilitates the comprehension of sentences in which such concept is employed metaphorically (e.g., *John grasps the idea*). Similar results (e.g., Boroditsky and Ramscar, 2002; Ackerman et al., 2010; Santana and de Vega, 2011; Gibbs, 2013) can be taken to suggest that sensory-motor simulations subserve the comprehension of metaphorical expressions derived from conceptual metaphors. This conclusion is compatible with recent brain imaging studies. Boulenger et al. (2009) found that both literal and figurative action sentences involving verbs related to the leg and arm elicited somatotopic activation. Lacey et al. (2012) used functional magnetic resonance imaging (fMRI) to demonstrate that texture-selective somatosensory cortex in the parietal operculum is more activated when processing expressions containing texture-based metaphors (*She has a rough day*), as compared to literal sentences matched for meaning. In this vein, it follows that individuals who had not been exposed to the sensory-motor domain in which conceptual metaphors are grounded should face serious limitations for comprehending metaphorical expressions, to the extent that their interpretation requires real-time simulations of such sensory-motor domains.

According to Lakoff and Johnson (1980) and Gibbs (2006b), expressions such as *There are some obscure points in the theory* and *This is an illuminating paper on the subject* constitute evidence of a primary conceptual metaphor, by which the activity of comprehending is understood in terms of visual concepts. This way, in an expression such as *A closer look at the idea will better reveal its details* the concept of comprehending an idea in more detail could only be understood in terms of recreating the sensory-motor experience of getting closer to an object, with the consequent enhancement in its visual definition. From this it follows that the congenitally blind should experience serious difficulties in making sense of metaphorical expressions derived from the UNDERSTANDING IS SEEING conceptual metaphor.

In contrast to this account, theoretical proposals not committed to the modal grounding of conceptual metaphors lead to a more optimistic perspective for the possibility that congenitally blind participants might comprehend visual metaphors. In line with traditional positions in cognitive science, several theories of analogy (e.g., Gentner’s structure-mapping theory; see Bowdle and Gentner, 2005) assume that both the source and the target domains of conceptual metaphors are mentally represented in the form of propositions (the basic units of the “language of thought”; Fodor, 1975), composed of amodal symbols. Hence, they conceive metaphor as the projection of amodal base concepts (referred to sensory-motor experiences but not isomorphic to them) onto amodal representations of abstract concepts.

As an illustration of how analogy theories apply to metaphor, consider Virginia Woolf’s metaphor *She allowed life to waste like a tap left running* (derived from the LIFE IS A VALUABLE RESOURCE and TIME IS A SUBSTANCE THAT FLOWS conceptual metaphors) as discussed by Gentner et al. (1988). Faced with this metaphorical expression, the interpreter is assumed to start off with some notion of water flowing through a tap into a drain [FLOW (water, tap, drain)] with the consequent disappearance of the water through the drain [DISAPPEAR (water, drain)], as well as with the idea that waste occurs if an agent allows such a flow to occur with no purpose, as in CAUSE [WITHOUT-PURPOSE (FLOW (agent, tap, water), WASTE (agent, water)]. It is also assumed that the interpreter has the notion that life flows from present to past [FLOW (life, present, past)] with the consequent disappearance of life into the past [DISAPPEAR (life, past)], as well as the additional proposition that the protagonist’s life is being wasted: WASTE (protagonist, life). As implemented in Structure Mapping Engine (SME, Falkenhainer et al., 1989), the cognitive system begins by finding *local matches*—potential matches between single items in the base and the target. For each entity and predicate in the base, it finds the set of entities or predicates in the target that could plausibly match that item. These potential match hypotheses are determined by pairing identical relations (e.g., FLOW↔FLOW), checking whether the arguments of the paired relations are formally identical (e.g., agents, patients, instruments, etc.), and pairing such propositional elements according to their roles within the proposition (e.g., water↔life, tap↔present, drain↔past). Due to the fact that the complete collection of local matches may be inconsistent, the cognitive system disambiguates inconsistencies by granting higher evidence scores when the paired relations are each subordinate to higher order relations which are paired as well (*systematicity principle*), as is reflected here by the fact that the flow of water causes it to disappear through the drain, just as the flow of life causes it to disappear into the past. In a typical analogical comparison, unpaired base assertions connected to global mappings are brought over to the target domain as candidate inferences (e.g., the system brings across the inference that the protagonist is letting her life pass with no purpose, and that this purposeless flow is causing her life to be wasted).

Given that analogical mapping and inference critically dependent on variable binding, it has been postulated that models that lack this key computational property (i.e., non-propositional models) may not be able to capture analogical thinking (Doumas and Hummel, 2005). The assumption that propositional representations constitute the only medium capable of supporting the comprehension, production and extension of analogical comparisons, together with the idea that conceptual metaphors consist of large-scale analogical mappings, lead directly to the conclusion that they are *necessarily* encoded in propositional format.

According to this perspective, interpreting a visual metaphor such as *His ideas were so luminous that they dazzled our* *reason* involves applying non-analogical symbols referred to the annulation of sight in order to understand other abstract concepts referred to the possibility of assessing someone else’s ideas in a proper way. An implication that follows from this non-experiential account of conceptual metaphors is that individuals could conceivably make sense of utterances derived from a conceptual metaphor even if they had not acquired direct sensory-motor experience with the base concepts to which the target domain is metaphorically associated. In the particular case of congenital blindness, the extent to which blind participants will be capable of understanding visual metaphors would depend critically on the richness and precision of their (amodal) representations of visual concepts, as well as on their ability to comprehend metaphors in general.

The hypothesis that blind individuals might succeed in understanding visual metaphors is not only compatible with classical amodal positions in cognitive science, but also with more recent hybrid proposals, in terms of which modal analogical representations coexist with amodal symbols hosted in brain locations that are close to perceptual areas. The first source of evidence for this idea involves neuropsychological patients with disorders such as semantic dementia, a pathology characterized by a bilateral atrophy of the temporal lobes, with progressive loss of semantic memory for common objects. The concomitant degradation of semantic knowledge often proceeds in a hierarchical fashion. For example, patients gradually lose the ability to identify more and more bird species, but remain able to classify most of them as birds or animals (Hodges et al., 1995; Patterson et al., 2007). With regards to neurotypical participants, recent research shows that transcranial magnetic stimulation of temporal areas leads to decreased efficiency with respect to semantic-processing tasks (Pobric et al., 2010). Although this decreased performance is far less catastrophic than the impairments found among patients with semantic dementia, it also implicates the anterior temporal lobes in semantic processing. These findings are often seen as either a vindication of classic amodal positions or as demonstration of the need for a hybrid approach. A particular class of intermediate theories expand on the notion of convergence zones (Damasio and Damasio, 1994), positing amodal hubs that radiate to modality-specific spokes in sensory-motor areas (Patterson et al., 2007; Lambon Ralph et al., 2009). An influential example of these hub-and-spoke theories locates a core, amodal hub for conceptual processing bilaterally in the anterior temporal lobes (Lambon Ralph et al., 2009; Pobric et al., 2010). Related theories have postulated the existence of several hubs and offer a more dynamic view of the interaction between amodal and modality-specific systems (e.g., Binder and Desai, 2011; Watson and Chatterjee, 2011; Reilly et al., 2014).

Consistent with evidence for the existence of amodal areas, some studies have failed to document differences between blind and sighted populations with respect to their literal understanding of visual language (e.g., *I* *see* or *Let me show you*), as well as of action verbs that convey visual motion (e.g., *run*) (e.g., Demott, 1972; Marmor, 1978; von Tetzchner and Martinsen, 1980; Landau and Gleitman, 1985; Rosel et al., 2005; Bedny et al., 2012; Dimitrova-Radojichikj, 2015). However, a handful of studies have detected differences in several aspects related to color terms (e.g., Shepard and Cooper, 1992; Geld and Stanojević, 2006; Geld and Starčević, 2006) and many other visual concepts such as *transparency* or *perspective* (Blagden and Everett, 1992; Marek, 1999). In light of these mixed results, the question arises as to whether the knowledge that blind people possess about the literal meaning of visual words is sufficient to support the metaphorical use of those terms.

In favor of the idea that blind’s knowledge of literal visual terms might enable their metaphorical projection, most accounts of metaphor processing posit that the metaphoric use of a base concept typically involves highlighting some abstract relational pattern over concrete particularities, in order to increase its scope (Jamrozik et al., 2016; Gentner and Asmuth, 2017). For instance, while the ability to understand or employ the literal concept of *moved photograph* in an accurate way would demand having observed such kind of images (e.g., so as to differentiate it from the murkiness caused by a dirty lens), comprehending a metaphorical expression such as *The report presents a moved photo of the real situation* might only require extracting a coarser meaning of the base concept (e.g., as involving “an imprecise representation”), something that could potentially be afforded by an amodal representation of the concept. In sum, it is at least conceivable that the superiority that sighted participants can exhibit with regards to the comprehension of literal visual concepts may on occasions involve a level of detail that exceeds what is required to understand their metaphorical applications.

In support of the hypothesis that modal information may not be required for the metaphorical application of perceptual concepts, several studies have shown that metaphorical use of perceptual concepts elicits activation of motor areas to a lesser degree than its literal use. For example, Aziz-Zadeh et al. (2006) analyzed the effector-specific brain activation elicited by action words embedded in either literal (e.g., *Biting a banana*) or metaphorical sentences (e.g., *Handling the truth*). Whereas literal sentences gave rise to differential activations in the premotor cortex as a function of whether action verbs involved the hand, the foot or the mouth, no such effector-specific activations were found among the corresponding metaphorical expressions. In a related study, Desai et al. (2013) compared the neural responses to sentences in which the same verb was used to express either literal action (*The daughter grasped the flowers*), metaphoric action (*The public grasped the idea*), idiomatic action (*The congress is grasping at straws in the crisis*) or abstract action (*The public understood the idea*). The authors observed that the activation of the anterior inferior parietal lobule—an area known to be associated with processing internal representations of movements and body part positions, integrating object knowledge and body representations—was higher for literal than for metaphorical expressions and abstract expressions, suggesting a role for high-level action simulations. While metaphorical expressions also activated the aIPL, they did not involve primary motor or motion-related areas, a result that was interpreted by the authors as fitting with the proposal that our understanding of metaphors depends in part on amodal systems.

Experiment 1 was carried out to assess whether congenitally blind participants achieve a less appropriate comprehension of visual metaphors, as predicted by conceptual metaphor theory, or whether their knowledge of literal visual concepts enables them to understand visual metaphorical expressions on par with their sighted peers.

## Experiment 1

### Method

#### Participants

Twenty congenitally blind (12 female, mean age = 26.15 years, *SD* = 4.65) and 20 sighted participants (10 female, mean age = 18.7 years, *SD* = 0.73) took part in the experiment for a stipend. All of them were native speakers of Spanish, and were first and second year undergraduates at major Argentine universities. The causes of blindness included retinopathy of prematurity (8), congenital glaucoma (4), optic nerve abnormalities (5) and Leber’s amaurosis (three). All participants signed an informed consent for participation in the study in accordance with the Declaration of Helsinki.

#### Materials

All participants listened to a set of 30 short (<5 words) metaphorical expressions comprising 10 expressions in which phenomena related to the domain of understanding were expressed in visual terms (e.g., *Clear book*; henceforth *visual metaphorical expressions*), 10 expressions in which the domain of understanding was conveyed in terms of grasping (e.g., *Handle an idea*; henceforth *grasping metaphorical expressions*) and 10 metaphorical expressions not based on culturally shared conceptual metaphors (e.g., *turtle employee*; henceforth *other metaphorical expressions*). **Appendix A** lists the original visual expressions in Spanish, together with their English translation. While the latter type of metaphorical expressions served as a control for whether blind and sighed participants were similarly able to comprehend metaphorical language in general, grasping metaphorical expressions served to assess whether both populations were equally capable of understanding metaphorical expressions derived from another primary conceptual metaphor applicable to the concept of comprehending. Each metaphor was followed by a multiple choice question including four options: its most appropriate meaning, two defensible but less precise interpretations of its meaning, and an alternative for when the participant could not make sense of the expression. As an example, the target expression *Clear book* was followed by (a) book that is easy to comprehend, (b) clever book, (c) attractive book, and (d) the expression doesn’t make sense to me. Stimuli only included sets of materials for which four experts in Language and Literature agreed on which option represented the most appropriate interpretation of the metaphorical expression.

#### Procedure

The computer-based procedure was programmed on DMDX software (Forster and Forster, 2003). The administration was self-paced, and involved groups of three or less participants working individually. During a brief instructional phase, participants of both groups were told that they would receive several metaphors—each one followed by four answer options—, and that they would have to choose the option that best conveyed the meaning of each metaphor. They were told that they could listen to the metaphors and either option as many times as they needed and that they could advance (return) to a subsequent (previous) sentence by using the right (left) arrow keys, or replay the last heard sentence using the downward key. They should select one option by pressing the upward key immediately after such option was played. Once an answer for a set was provided, they heard “Now you can begin with the following metaphor.” Participants could navigate back and forth between a current metaphor and its options, but the system did not allow them to revisit a previous set after its corresponding answer was confirmed. While target sentences were preceded by the words “metaphor,” answer options “a” to “d” were preceded, respectively, by the words “option A,” “option B,” “option C” and “option D.” After four practice trials, participants received the metaphorical expressions in counterbalanced order. The presentation of the answer options was also counterbalanced, except for the option “The expression doesn’t make sense to me,” which was always presented last. Participants took an average of 45 min to complete the experiment.

### Results and Discussion

**Table 1** displays the mean proportion of correct answers provided by blind and sighted participants for each group of metaphorical expressions. A 2 × 3 mixed analysis of variance (ANOVA) with visual condition (blind vs. sighted) as between-subjects factor and type of metaphorical expression (visual, grasping, and other) as within-subjects factor was carried out to reveal the effects of these variables on participants’ comprehension of metaphorical expressions (Greenhouse–Geisser correction was applied do to a lack of sphericity in the data). Main effects were neither found for type of metaphorical expression, *F*(1.65,62.80) = 0.292, *MSE* = 0.018, *p* = 0.706, $\eta_{\text{p}}^{2}$ = 0.008, nor for visual condition, *F*(1,38) = 0.093, *MSE* = 0.014, *p* = 0.762, $\eta_{\text{p}}^{2}$ = 0.002. The interaction between these factors was not significant, *F*(1.65,62.80) = 0.360, *MSE* = 0.014, *p* = 0.659, $\eta_{\text{p}}^{2}$ = 0.009. These data seems to indicate that congenitally blind participants are comparable to their sighted peers both at comprehending metaphorical language in general and in making sense of other non-visual metaphorical expressions referred to the target concept of understanding (derived from an alternative primary conceptual metaphor). More strikingly, they equaled their sighted peers at making sense of metaphorical expressions derived from the UNDERSTANDING IS SEEING conceptual metaphor.

**Table 1:** Mean proportions of correct answers for the comprehension of metaphorical expressions by blind and sighted participants, Experiment 1.

	Blind participants		Sighted participants	
	*M*	*SD*	*M*	*SD*
Seeing metaphorical expressions	0.84	0.08	0.86	0.14
Grasping metaphorical expressions	0.85	0.08	0.83	0.14
Other metaphorical expressions	0.84	0.14	0.82	0.13

It should be noted, however, that most of the metaphorical expressions used as critical stimuli were rather conventional, with frequencies of occurrence in the “Google” corpus higher than 500 hits. Given the average conventionality of visual expressions (e.g., “obscure” means “difficult to understand”), blind participants might have based their interpretation on a second sense of such metaphorical concepts, a meaning which was already available in their mental lexicon and which may have already lost its link with its corresponding literal visual sense (Glucksberg, 2001; Bowdle and Gentner, 2005). It could be the case that, as opposed to understanding conventional expressions, making sense of novel metaphorical expressions makes it unavoidable to access the literal meaning of base concepts and, according to an embodied version of the career of metaphor thesis, even to recruit the sensory-motor experiences that characterize the base domain (the neural career of metaphor, Desai et al., 2011). Experiment 2 was carried out to assess whether congenitally blind participants are on par with their sighted peers in their comprehension of novel metaphorical expressions derived from the UNDERSTANDING IS SEEING conceptual metaphor, or whether they show a more limited understanding of this type of expressions.

## Experiment 2

### Method

#### Participants

Twenty congenitally blind (10 female, mean age = 27.2 years, *SD* = 3.88) and 20 sighted participants (12 female, mean age = 18.85 years, *SD* = 0.67) took part in the experiment for a stipend. All of them were native speakers of Spanish, and were first and second year undergraduates at major Argentine universities. The causes of blindness included retinopathy of prematurity (9), congenital glaucoma (6), and optic nerve abnormalities (5). All subjects signed an informed consent for participation in the study in accordance with the Declaration of Helsinki. No participants of Experiment 2 had previously participated in Experiment 1.

#### Materials and Procedure

As in Experiment 1, participants listened to a set of 30 short (<5 words) sentences comprising 10 visual metaphorical expressions, 10 grasping metaphorical expressions, and 10 other metaphorical expressions not derived from conceptual metaphors. **Appendix B** lists the original visual expressions in Spanish, together with their English translation. All metaphorical expressions as well as their slight modifications showed no or extremely low (<5 hits) occurrence in the “Google” corpus. As in Experiment 1, each metaphorical expression was followed by a multiple choice question including four options: its most appropriate meaning, two defensible but less precise expressions of its meaning, and a “the expression doesn’t make sense to me” option. As in Experiment 1, stimuli only included sets of materials for which four experts in Language and Literature agreed on which option represented the most appropriate interpretation of the metaphorical expression. The procedure followed with blind and sighted participants was identical to that of Experiment 1.

### Results and Discussion

**Table 2** displays the mean proportion of correct answers provided by blind and sighted participants for each group of novel metaphorical expressions. A 2 × 3 mixed analysis of variance (ANOVA) with visual condition (blind vs. sighted) as between-subjects factor and type of metaphorical expression (visual, grasping, and other) as within-subjects factor was carried out to reveal the effects of these variables on the comprehension of novel metaphorical expressions. Main effects were neither found for type of metaphorical expression, *F*(2,76) = 1.845, *MSE* = 0.004, *p* = 0.165, $\eta_{\text{p}}^{2}$ = 0.046, nor for visual condition, *F*(1,38) = 0.002, *MSE* = 0.052, *p* = 0.968, $\eta_{\text{p}}^{2}$ = 0.000. The interaction between these variables was not significant, *F*(2,76) = 0.475, *MSE* = 0.052, *p* = 0.623, $\eta_{\text{p}}^{2}$ = 0.012.

**Table 2:** Mean proportions of correct answers for the comprehension of metaphorical expressions by blind and sighted participants, Experiment 2.

	Blind participants		Sighted participants	
	*M*	*SD*	*M*	*SD*
Seeing metaphorical expressions	0.72	0.16	0.71	0.12
Grasping metaphorical expressions	0.74	0.13	0.73	0.14
Other metaphorical expressions	0.70	0.16	0.71	0.13

These data reveal that congenitally blind participants are comparable to their sighted peers both at comprehending novel metaphorical expressions in general and in making sense of non-visual metaphorical expressions referred to the target concept of understanding. Despite a lack of experience with the visual domain, blind participants also equaled their sighted peers at making sense of metaphorical expressions derived from the UNDERSTANDING IS SEEING conceptual metaphor.

## General Discussion

From the embodied perspective of conceptual metaphor theory, it follows that congenitally blind participants might face serious difficulties for making sense of visual metaphors, on the grounds that their comprehension requires real-time simulations of the sensory-motor experiences on which they are grounded. In contrast to an embodied account, dominant theories of analogical thinking have conceived of metaphorical processing as involving propositional structures that combine amodal representations of concepts, a position that is also compatible with recent hybrid models of concept representation. From this perspective, blind participants could conceivably make sense of visual metaphors to the extent that their (amodal) literal representations of visual concepts include the essential information that is required to support their metaphorical application.

Experiment 1 failed to detect group differences in understanding this particular kind of expressions. Given the rather conventional nature of the metaphors employed in Experiment 1, it could be argued that blind participants could have interpreted these utterances by way of accessing a second meaning of the metaphorical terms employed in such expressions, one that may have lost its connection with the literal sense from which they were derived (see, e.g., Glucksberg, 2001; Bowdle and Gentner, 2005). With this possibility in mind, in Experiment 2 we presented blind and sighted participants with novel visual metaphors that had either low or no occurrences in the “Google” corpus. Despite the novelty of these metaphorical expressions, blind participants showed a comprehension that was not only accurate in absolute terms, but also similar to that of their sighted peers. One possible way to make sense of this finding would consist in positing that despite the minimal frequency of novel metaphorical expressions, their interpretation rests on well-rehearsed mechanisms for extending visual concepts to the domain of comprehension. Given that there are numerous high-frequency visual metaphors for the target domain of understanding, it is conceivable that both blind and sighted populations are familiar with those aspects of the base domain that are recurrently selected to be projected onto the domain of understanding. Hence, interpreting metaphorical expressions with low or no frequency could conceivably be easy even for blind participants, as long as those metaphors appeal to aspects of the base domain that are recurrently projected onto the target. To exemplify, when interpreting a rather conventional expression like *A maquillaged version of reality* we can either access a second meaning of *maquillage* or a pre-stored representation of this particular metaphorical expression. But if someone now utters a completely novel expression such as *A photoshopped version of reality*, making sense of such sentence as implying a biased rendition of reality might not be significantly more demanding than in the former case, since the relevant meaning of *photoshopped* in this context can easily be derived. To a great extent, conceptual metaphors are universal knowledge structures that are associated to the structure of our body and of our exchanges with the world. As such, they impose strong constraints on the space of possible innovations in metaphoric production. These considerations can shed new light to intuitions like those of Jorge Luis Borges, who asserted that: “though there are hundreds and indeed thousands of metaphors to be found, they may all be traced back to a few simple patterns” (Borges, 1967/2000, p. 40). Future studies should assess whether comprehending metaphors that exploit rare or infrequent aspects of the base domain might require the availability of modal representations.

Even though the ideas that led to the hypothesis that blind participants could conceivably understand visual metaphors stem from with an amodal account of how literal concepts and conceptual metaphors are represented (e.g., with the propositional model of Bowdle and Gentner, 2005), the comprehension capabilities exhibited by blind participants cannot be conclusively taken to confirm an amodal account of how these conceptual structures are encoded and processed. From an embodied perspective, it could be argued that blind participants make sense of visual metaphorical expressions via *non-visual* sensory-motor simulations. In support of this idea, a long tradition exploring the imagery capabilities of congenitally blind individual (e.g., Kerr, 1983; Zimler and Keenan, 1983; see Kaski, 2002, for a review) had suggested that they can handle mental representations that preserve spatial aspects of reality such as distance and occlusion. Hence, blind participants could make sense of an utterance like *A dense cloud obscured his ideas* by activating a spatial version of the COMPREHENDING IS SEEING conceptual metaphor, in terms of which a tangible entity (e.g., a piece of cloth) is interposed between the observer and an object that would otherwise be reachable. Similarly, it is possible that for metaphorical expressions such as *A closer look at the idea will reveal its imperfections*, blind participants may have learned (see, e.g., Landau and Gleitman, 1985) how to perform experiential “translations” of metaphorically visual concepts in terms of concepts related to touching or grasping, and thus, to simulate visual metaphors in these terms (e.g., they could simulate the referred expression as an act of slowly probing a surface so as to reveal its subtle rugosities). Despite the plausibility of this account for rather conventional visual metaphors as those exemplified above, it is not easy to foresee how it could be extended to explain the interpretation of some of the novel expressions included in Experiment 2, whose visual terms resist an easy translation into purely spatial or haptic experiences. Further studies are required to determine which of these two approaches best accounts of how blind populations cope with making sense of visual metaphors. In turn, this line of inquiry will benefit from achieving a deeper understanding of how the blind acquire and represent the literal meaning of visual concepts. One promising avenue for research could consist in employing brain imaging techniques to assess whether the literal vs. metaphoric use of visual concepts by congenitally blind participants can be accommodated by the hybrid models recently proposed, as well as whether the patterns of brain activation elicited by these expressions differ between blind and sighted participants. Even though the present results indicate that these populations are equally able to comprehend conventional as well as novel visual metaphors, we see no reasons to assume that both populations will base their comprehension of these expressions on an amodal substrate: whereas blind participants would necessarily employ amodal symbols, sighted participants could conceivably recruit both kinds of representations.

The present investigation represents an extreme exemplar of a broader tradition of studies finding that blind individuals manage to match their sighted peers on tasks and activities for which intuitive considerations might lead to anticipate a relative disadvantage. Advancing our understanding of how blind individuals make sense of visual metaphors will contribute to a broader understanding of how the blind as well as other sensorily deprived populations overcome significant limitations for acquiring knowledge about their environment.

## Data Availability Statement

The raw data supporting the conclusions of this manuscript will be made available by the authors, without undue reservation, to any qualified researcher.

## Author Contributions

RM designed the studies, administered the experiment, and participated in the writing of the manuscript. AM programmed the computer interface and collaborated in the administration of the experiments. LT participated in the data analysis and literature review. MT participated in the design of the study and the writing of the paper.

## Conflict of Interest Statement

The authors declare that the research was conducted in the absence of any commercial or financial relationships that could be construed as a potential conflict of interest.

## Figures and Tables

**Table:** 

Original Spanish expression	English translation
1. Libro claro	Clear book
2. Teoría oscura	Obscure theory
3. Distinción borrosa	Blurry distinction
4. Cambiar de punto de vista	Change of point of view
5. Interpretación brillante	Brilliant interpretation
6. Entrever una dificultad	Glimpse a difficulty
7. Explicación transparente	Transparent explanation
8. Idea maquillada	Maquillaged idea
9. Descripción panorámica	Panoramic description
10. Análisis miope	Myopic analysis

**Table:** 

Original Spanish expression	English translation
1. Ecografía de sus pensamientos	Echography of her thoughts
2. Comprensión en 3D del fenómeno	3D comprehension of the phenomenon
3. Foto movida de la realidad	Moved photograph of reality
4. Comentario laser	Laser comment
5. Hechos fotoshopeados	Photoshopped facts
6. Conclusión fluorescente	Fluorescent conclusion
7. Ideas desteñidas	Off-colored ideas
8. Mente titilante	Titling mind
9. Teoría translúcida	Translucent theory
10. Entendimiento con luces de posición	Understanding with parking lights

## Appendix A

Visual metaphorical expressions used in Experiment 1, along with their English translations.

## Appendix B

Visual metaphorical expressions used in Experiment 2, along with their English translations.
